# miR-205-5p drives endothelial dysfunction and senescence in pulmonary fibrosis

**DOI:** 10.1172/jci.insight.201842

**Published:** 2026-05-28

**Authors:** Giuseppe Muscato, Benjamin B. Roos, Sharonda Harris, Xiaoyu Tracy Cai, Gina Civettini, Enrico Sciacca, Ahmed A. Raslan, Alessandra Castaldi, Sharon Elliot, Marilyn K. Glassberg, Carlo Vancheri, Daniel J. Tschumperlin, Giovanni Ligresti, Nunzia Caporarello

**Affiliations:** 1 Department of Clinical and Experimental Medicine, University of Catania, Catania, Italy.; 2 Department of Medicine, Stritch School of Medicine, and; 3 Department of Cell and Molecular Physiology, Loyola University Chicago, Maywood, Illinois, USA.; 4 Department of Medicine, Boston University, Chobanian and Avedisian School of Medicine, Boston, Massachusetts, USA.; 5 Department of Zoology, Faculty of Science, Assiut University, Assiut, Egypt.; 6 Department of Medicine, University of California, San Diego, La Jolla, California, USA.; 7 Department of Physiology and Biomedical Engineering, Mayo Clinic, Rochester, Minnesota, USA.

**Keywords:** Pulmonology, Vascular biology, Endothelial cells, Fibrosis, Noncoding RNAs

## Abstract

Idiopathic pulmonary fibrosis (IPF) is a fatal, aging-related disease characterized by persistent lung fibroblast activation, progressive lung scarring, and several vascular abnormalities. We have previously demonstrated that aging-associated vascular dysfunction drives maladaptive endothelial responses to injury and exacerbates lung fibrosis via secretion of profibrotic endothelial cell–derived factors. However, regulatory mechanisms governing endothelial dysfunction during progressive lung fibrosis remain poorly understood. Here, using preclinical mouse models of progressive lung fibrosis as well as human IPF lungs, we demonstrate that miR-205-5p was overexpressed in lung endothelial cells (ECs) from fibrotic lungs and coordinated gene expression programs implicated in endothelial dysfunction and progressive fibrosis. Mechanistically, miR-205-5p induced senescence in lung ECs, mirroring the senescent phenotype of IPF lung ECs. Consistently, conditioned medium derived from lung ECs overexpressing miR-205-5p promoted lung fibroblast activation. Importantly, miR-205-5p inhibition in IPF lung ECs attenuated endothelial senescence and limited paracrine fibroblast activation. Finally, inhibition of miR-205-5p in vivo preserved the pulmonary vascular network and attenuated lung fibrosis progression in aged mice challenged with bleomycin. Collectively, our findings support what we believe to be a novel connection among lung endothelial miR-205-5p, endothelial senescence, and profibrotic alteration of the endothelial secretome and highlight miR-205-5p inhibition as a potential therapeutic intervention for pulmonary fibrosis.

## Abstract 2

This study identifies miR-205-5p as a key regulator of blood vessel dysfunction and fibroblast activation in pulmonary fibrosis.

## Graphical abstract

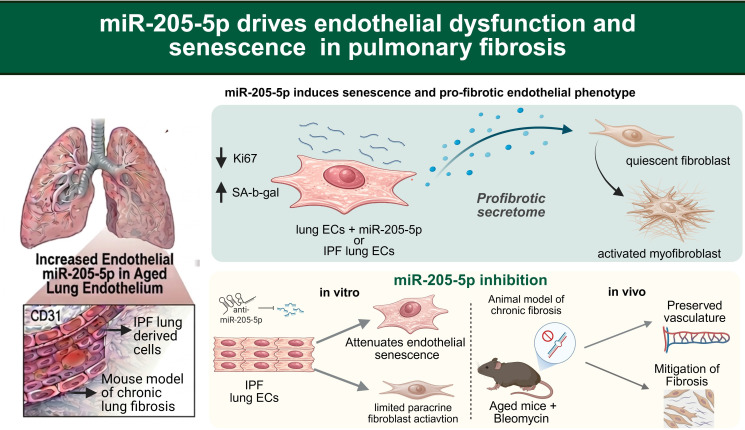

## Introduction

Idiopathic pulmonary fibrosis (IPF) is a progressive, aging-related disease characterized by persistent activation and sustained accumulation of scar-forming fibroblasts (1, 2). IPF arises from a complex network of interactions among different cell types and profibrotic signaling pathways, making therapeutic advancement particularly challenging. Despite the ability of existing therapies to attenuate lung functional decline, they fail to halt fibrotic progression, highlighting the persistent unmet clinical need in IPF (3). While prior work has largely focused on the role of mesenchymal, epithelial, and immune cell types in IPF pathogenesis and considered them as potential therapeutic targets, a growing body of evidence, including our own, supports the notion that endothelial abnormalities play a critical role in the progression of IPF (4–12). Recent work from our group has demonstrated that the pulmonary vasculature of young mice orchestrates reparative responses to lung injury by activating transcriptional programs promoting vascular repair and fibrosis resolution (7, 11). In contrast, the pulmonary vasculature of aged mice, after injury, loses this ability and exhibits transcriptional alterations resulting in the secretion of soluble profibrotic mediators, ultimately impairing fibrosis resolution. However, the mechanisms governing these maladaptive endothelial transcriptional responses to lung injury remain largely unclear. Given their capacity to modulate gene expression programs (13), microRNAs (miRNAs) are plausible candidates for influencing endothelial transcription and injury responses. Notably, miRNAs have been shown to regulate lung development and participate in the lung’s response to injury, and altered levels have been implicated in the progression of chronic lung diseases, including IPF (14). Yet, their specific contribution to endothelial dysfunction in IPF remains largely unknown. To explore endothelial miRNA changes associated with lung disrepair and persistent fibrosis, we performed a miRNA screen on lung endothelial cells (ECs) freshly isolated from aged mice with progressive fibrosis and identified increased expression of miR-205-5p. Subsequent analyses confirmed increased miR-205-5p expression in lung ECs isolated from young mice with persistent lung fibrosis induced by repetitive bleomycin injury and from human IPF lungs. In vitro studies demonstrated that miR-205-5p induces transcriptional changes in human lung ECs associated with cell cycle arrest and secretion of profibrotic mediators, including components of the senescence-associated secretory phenotype (SASP), while also inducing the expression of the senescence marker β-galactosidase, mirroring the phenotype of cultured lung ECs isolated from IPF lungs. Conditioned medium from miR-205-5p–overexpressing lung ECs promoted the transition of quiescent lung fibroblasts into activated fibroblasts. Integrated target prediction and network analysis identified YAP and CDKN1A signaling pathways as putative regulatory axes downstream of miR-205-5p in control lung ECs. Notably, inhibition of miR-205-5p in IPF lung ECs reduced the expression of genes associated with soluble profibrotic molecules belonging to the SASP, attenuated endothelial senescence, and limited paracrine fibroblast activation. Importantly, inhibition of miR-205-5p in aged mice challenged with bleomycin preserved the pulmonary vascular network and attenuated lung fibrosis progression.

Taken together, our findings demonstrate that miR-205-5p plays a crucial role in perpetuating lung endothelial dysfunction and promoting a profibrotic milieu driving progressive fibrosis. These data define a previously unrecognized miR-205-5p–dependent profibrotic endothelial state and suggest that its targeting may represent a potential therapeutic approach in lung fibrosis.

## Results

### Lung endothelial miR-205-5p is upregulated in preclinical mouse models of persistent lung fibrosis and in IPF lung ECs.

**Figure 1:**
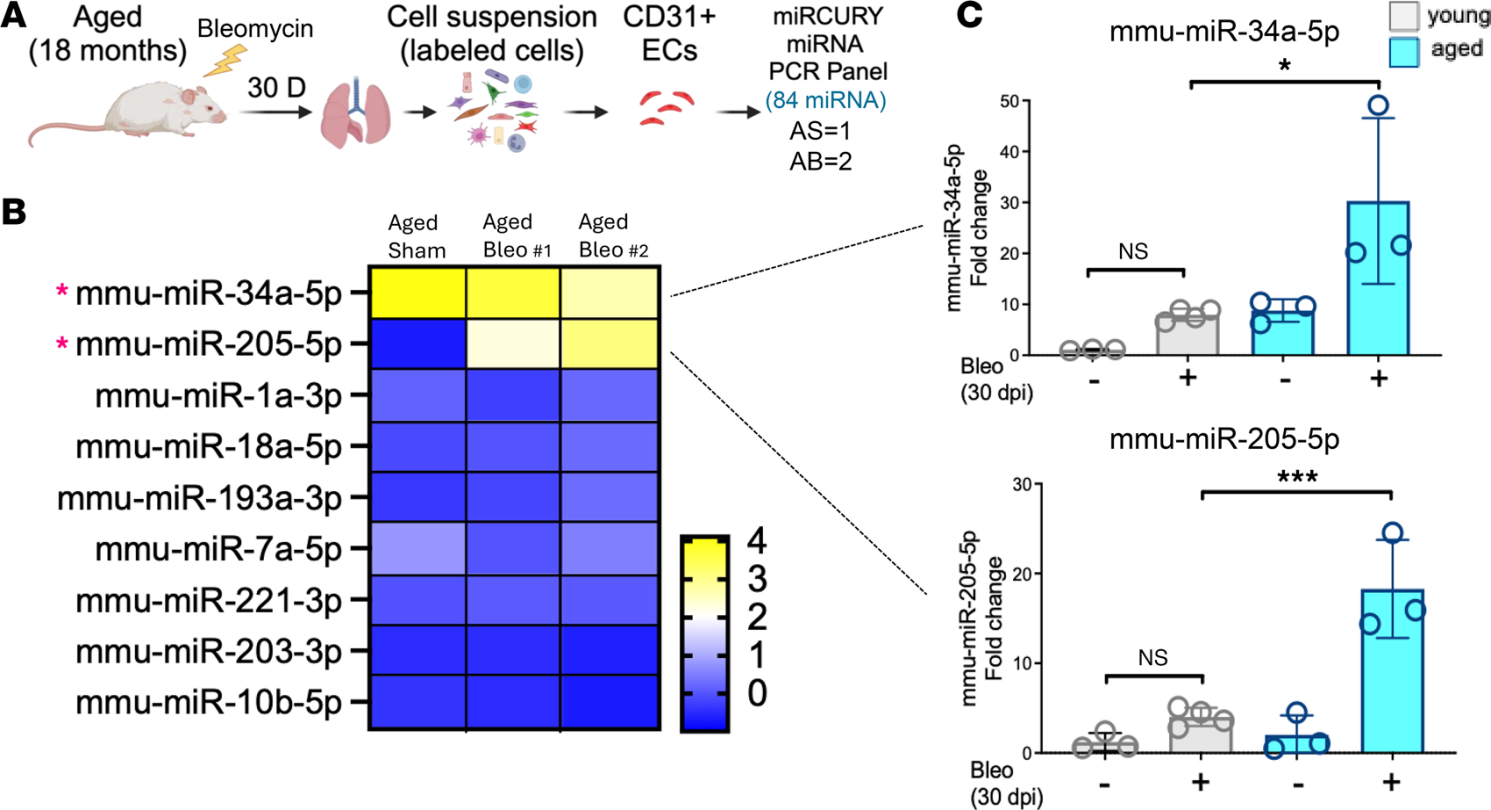
miRNA expression profile of lung ECs from aged mice with persistent lung fibrosis. (**A**) Experimental workflow: aged mice intratracheally received bleomycin (1.2 U/kg); lungs were collected on day 30 and dissociated into single cells, and CD31^+^ lung ECs were isolated by FACS (created with a licensed version of BioRender.com). (**B**) Endothelial miRNA expression was quantified using a miRNA focus qPCR panel including 84 miRNAs. Heatmap showing the top 9 differentially expressed miRNAs, with miR-205-5p and miR-34a-5p emerging as the most upregulated (Aged Sham, *n* = 1; Aged Bleo, *n* = 2, displayed as *z* score). (**C**) qPCR confirmed significant upregulation of miR-205-5p and miR-34a-5p in FACS-sorted CD31^+^ lung ECs from aged mice 30 days after bleomycin exposure compared with young mice under the same experimental condition. snU6 was used as normalization control. Data are represented as mean ± SD, and each dot represents an individual mouse (Young sham, *n* = 3; Young Bleo, *n* = 4; Aged sham, *n* = 3; Aged Bleo, *n* = 3). *P* values were calculated using 1-way ANOVA (followed by Tukey’s post hoc test). *P < 0.05; ***P < 0.001.

**Figure 2:**
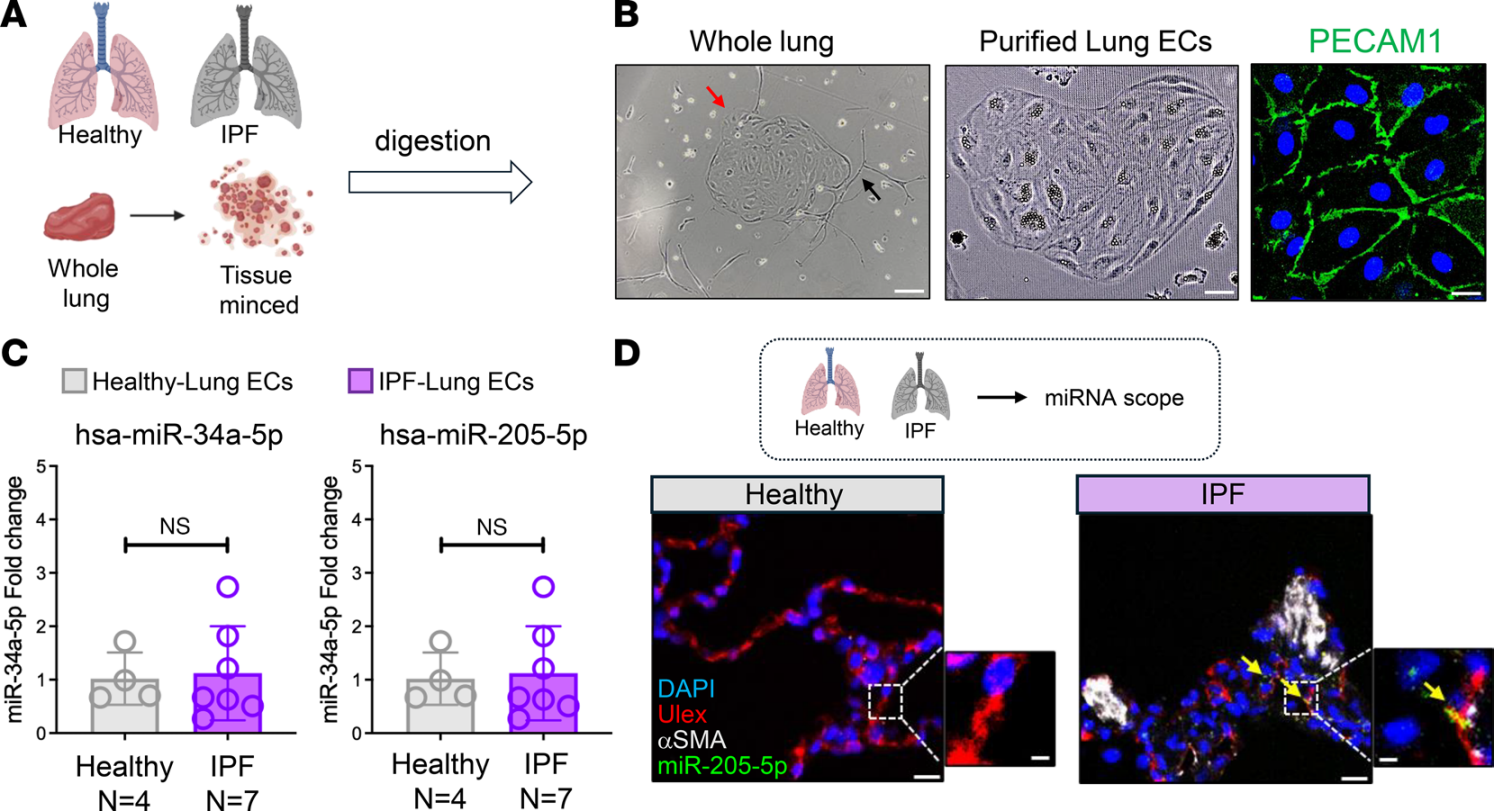
Lung endothelial miR-205-5p is overexpressed in IPF lung ECs and colocalizes with vessels near fibroblastic foci. (**A**) Schematic depicting our isolation protocol employed to purify lung ECs from control and IPF lungs (created with a licensed version of BioRender.com). (**B**) Purified lung ECs express the pan-endothelial marker PECAM1 (green). (**C**) qPCR shows higher levels of miR-205-5p in IPF lung ECs compared with healthy lung ECs, whereas no difference is observed in miR-34a-5p levels. snU6 was used as normalization control. Data are shown as mean ± SD (healthy, *n* = 4 donors; IPF, *n* = 7 patients), and *P* values were calculated using Student’s *t* test. (**D**) Representative combined immunofluorescence and miRNA Scope images of 1 healthy lung tissue and 1 IPF lung tissue. Ulex europaeus I lectin was used to stain vessels (red), αSMA antibody as marker of activated fibroblasts (white), miR-205-5p probe to detect human miR-205-5p, and DAPI for nuclei (blue). Scale bar: 50 μm.

Our published data documenting endothelial transcriptional changes in progressive lung fibrosis (7, 9, 11), combined with the concept that miRNAs play a pivotal role in controlling gene expression programs (15), led us to hypothesize that altered miRNAs levels may play a role in the pathogenic endothelial transcriptional programs associated with progressive lung fibrosis. To test this hypothesis, we carried out a preliminary miRNA expression screening analysis on FACS-sorted lung ECs isolated from aged mouse lungs with persistent fibrosis induced by bleomycin injury (Figure 1A). This analysis of 84 miRNAs revealed that several miRNAs are altered in lung ECs from aged lungs, with miR-205-5p and miR-34a-5p emerging as the highest expressed miRNAs (Figure 1B). Thus, we focused on miR-205-5p and miR-34a-5p and extended the analysis to a larger cohort of mice, including young and aged mice. As shown in Figure 1C, we found that miR-205-5p and miR-34a-5p were significantly increased during progressive lung fibrosis in aged mice. Interestingly, although levels of miR-205-5p and miR-34a-5p were slightly increased in young mice injured with bleomycin compared with uninjured young mice, these increases were not significant, and levels in young mice were significantly lower compared with aged mice after bleomycin (Figure 1C). To extend our findings to the context of human disease, we examined the expression of miR-205-5p and miR-34a-5p in lung ECs isolated from IPF lungs. Because these cells are not commercially available, we optimized our own method to isolate and culture these cells from IPF lungs with high purity, as demonstrated by immunofluorescence staining for the pan endothelial marker PECAM1 (IPF lungs obtained through Loyola University Transplant Center, Figure 2, A and B). Healthy lung ECs were isolated using the same method from donor lungs considered unsuitable for transplantation (donor lungs obtained through Gift of Hope). Intriguingly, qPCR analysis showed significantly higher levels of miR-205-5p levels in IPF lung ECs compared with healthy lung ECs, whereas miR-34a-5p levels were similar in both groups (Figure 2C), identifying miR-205-5p as a candidate regulator of lung endothelial dysfunction during progressive lung fibrosis both in mice and in humans. Intriguingly, miRNAScope staining combined with immunofluorescence showed negligible miR-205-5p expression in the vasculature of healthy human lung tissue, stained using Ulex europaeus I lectin, a marker for human blood vessel endothelium (16) (Figure 2D). In contrast, we found increased expression of miR-205-5p in IPF lung tissue (Figure 2D and Supplemental Figure 2; supplemental material available online with this article; https://doi.org/10.1172/jci.insight.201842DS1), particularly in vessels closely associated with novel foci of activated fibroblasts (Figure 2D). Next, to further explore the role of endothelial miR-205-5p in progressive fibrosis, we measured its endothelial levels in an additional model of progressive fibrosis, induced by repetitive bleomycin injury in young mice. Interestingly, we found that the expression of endothelial miR-205-5p was significantly upregulated in the repetitive injury model (Supplemental Figure 1), thus confirming lung endothelial miR-205-5p implication in progressive lung fibrosis. Based on these observations, we selected miR-205-5p as the candidate miRNA for our further studies.

### Lung endothelial miR-205-5p controls gene programs implicated in endothelial dysfunction and induces a prosenescent endothelial phenotype, mirroring IPF lung EC behavior.

**Figure 3:**
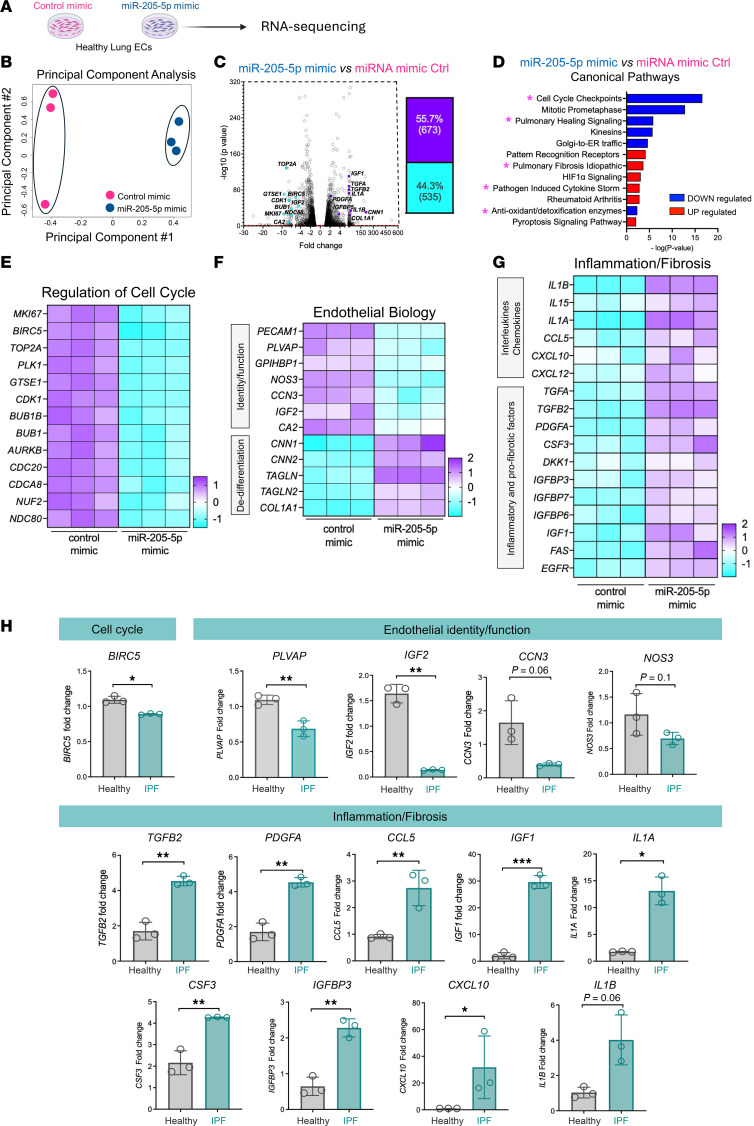
Lung endothelial miR-205-5p controls gene programs implicated in endothelial dysfunction and lung fibrosis, mimicking aberrations of IPF lung ECs. (**A**) Schematics depicting the experimental workflow. Healthy human lung ECs were transfected with negative control mimic or miR-205-5p mimic and profiled by RNA-seq (created with a licensed version of BioRender.com). (**B**) Principal component analysis (PCA) illustrating separation of experimental groups based on the differences in their transcriptomes. (**C**) Volcano plot of –log_10_(FDR *q*) versus log_2_(fold change) of genes with respect to miR-205-5p vs. mimic control; genes that are significantly (FDR *q* < 0.05) upregulated (>2-fold) or downregulated (<2-fold) in miR-205-5p–overexpressing lung ECs are indicated and tallied in purple and turquoise, respectively. (**D**) Ingenuity Pathway Analysis identifies differentially regulated canonical pathways; pink asterisks indicate pathways whose role in pulmonary fibrosis is known. (**E**–**G**) Heatmaps of differentially regulated genes, including genes responsible for cell cycle regulation (**E**), endothelial identity and function (**F**), and proinflammatory and profibrotic genes (**G**). Colors were assigned after *z* score–normalizing expression values to a mean of 0 and SD of 1 within each row, with turquoise, white, and purple indicating *z* scores ≤–2, 0, and ≥+2, respectively. (**H**) Bar plots showing fold change differences between IPF and healthy lung ECs for relevant genes involved in endothelial function and proinflammatory/fibrotic state. *N* = 3 independent biological replicates. Data are expressed as mean ± SD, and *P* values were calculated using Student’s *t* test. *P < 0.05; **P < 0.01; ***P < 0.001.

**Figure 4:**
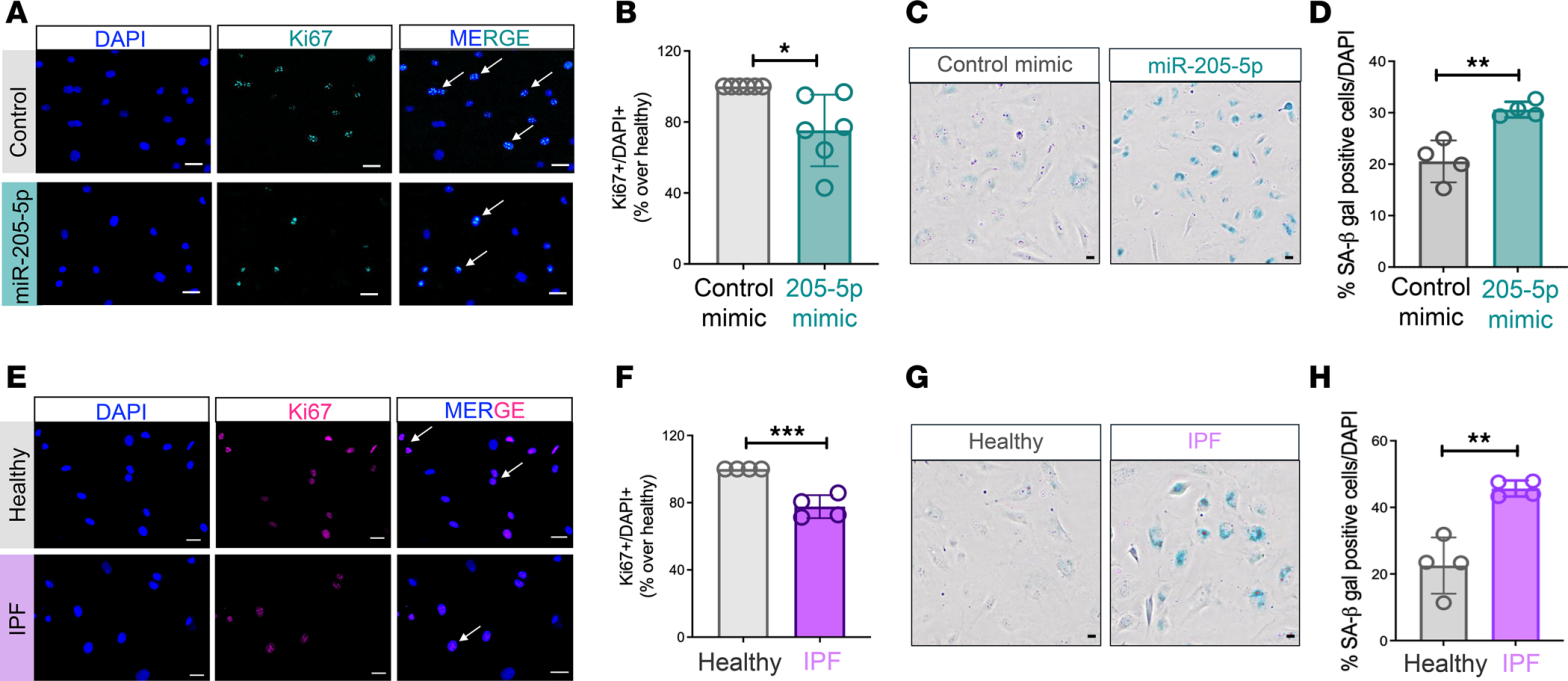
miR-205-5p induces senescence in lung ECs, mirroring cultured IPF lung ECs. (**A**) Representative immunofluorescence images for Ki67 and DAPI in human lung ECs transfected with a miRNA mimic negative control or miR-205-5p mimic. Arrows indicate Ki67^+^ nuclei. Scale bar: 20 μm. (**B**) Quantification of Ki67^+^ nuclei (normalized to DAPI) reveals a significant reduction in miR-205-5p mimic–transfected cells versus control cells. Data are shown as mean ± SD of *n* = 6 independent biological replicates (average of 3–5 images per replicate) and expressed as percentage over control. *P* values were calculated using Student’s *t* test. (**C**) Representative SA-β-gal staining images showing positive staining of miR-205-5p–overexpressing cells. Scale bar: 20 �m. (**D**) Quantification of SA-β-gal–positive cells (over total DAPI-positive cells) showing increase of SA-β-gal–positive cells in miR-205-5p–overexpressing lung ECs. Data are shown as mean ± SD of *n* = 4 independent biological replicates (average of 3–5 images per replicate), and *P* values were calculated using Student’s *t* test. (**E**) Representative immunofluorescence images for Ki67 and DAPI in healthy and IPF lung ECs. Arrows indicate Ki67^+^ nuclei. Scale bar: 20 μm. (**F**) Quantification of Ki67^+^ cells normalized to DAPI shows IPF ECs manifesting significant reduced Ki67 staining compared with healthy lung ECs. Data are shown as mean ± SD of *n* = 4 independent biological replicates (average of 3–5 images per replicate) and expressed as percentage over control. *P* values were calculated using Student’s *t* test. (**G**) Representative SA-β-gal staining images showing positive staining in IPF lung ECs. Scale bar: 20 �m. (**H**) Quantification of SA-β-gal–positive cells (over total DAPI-positive cells) showing increase of SA-β-gal–positive cells in IPF lung ECs. Data are shown as mean ± SD of *n* = 4 independent biological replicates (average of 3–5 images per replicate), and *P* values were calculated using Student’s *t* test. *P < 0.05; **P < 0.001.

To investigate the broad contribution of miR-205-5p in modulating the transcriptional landscape of lung ECs and its potential association with the aberrant endothelial phenotype in IPF, we used RNA-seq to profile gene expression in healthy lung ECs that were transfected with a miR-205-5p mimic or a negative control mimic (Figure 3A). Principal component analysis (PCA) revealed that samples clustered in 2 groups according to treatment (control vs. miR-205-5p overexpression) (Figure 3B). In comparing the transcriptomes of the 2 groups, we identified 1,208 differentially regulated genes, of which 55.7% were upregulated and 44.3% were downregulated (Figure 3C). Survival and cell cycle progression genes, including *BIRC5* and *TOP2A* were significantly downregulated by miR-205-5p overexpression (Figure 3C). In contrast, endothelial dedifferentiation genes and inflammation/fibrosis genes, including *CNN1*, *TGFB2*, *IL1B*, *IGF1*, and *IL1A*, were significantly upregulated in lung ECs overexpressing miR-205-5p compared with control cells (Figure 3C). Ingenuity Pathway Analysis (IPA) showed that most pathways that were downregulated by miR-205-5p overexpression were associated with cell cycle checkpoint, pulmonary healing signaling pathways, and regulation of antioxidant/detoxification enzymes (Figure 3D). Our analysis also led to the identification of pathways that were positively regulated by miR-205-5p overexpression, and among them were those related to HIF1α signaling and pathogen-induced cytokine storm signaling pathway (Figure 3D). To illustrate and compare transcriptional changes, we generated heatmaps of genes that are representative of signaling pathways implicated in EC biology and lung fibrosis that were regulated by miR-205-5p overexpression. We found that miR-205-5p downregulated several genes related to cell cycle progression, including *MKI67* (Figure 3E). Genes associated with endothelial identity and function, such as *PECAM1*, *PLVAP*, *GPIHBP1*, and *IGF2*, as well as *NOS3* and *CCN3*, both previously shown to ameliorate vascular aging and persistent fibrosis by our group (5, 9), were also downregulated (Figure 3F). In contrast, genes associated with endothelial dedifferentiation, including *CNN2* and *TAGLN*, were upregulated in miR-205-5p–overexpressing lung ECs. Moreover, genes encoding inflammatory/fibrotic secreted factors were upregulated by miR-205-5p (Figure 3G). Among these, *TGFB2* (17), *PDGFA* (18), *CSF3* (19), *CCL5* (20), *IGF1* (21), *IGFBP3* (22), and *IL1B* (23), encoding potent secreted inducers of lung fibroblast activation in IPF lungs and components of the SASP (24), were elevated. Collectively, these data implicate miR-205-5p in the regulation of cell cycle arrest, endothelial dedifferentiation, and proinflammatory/profibrotic remodeling of the endothelial secretome. Intriguingly, a similar pattern of gene expression was observed in cultured IPF lung ECs (Figure 3H), with downregulation of survival- and endothelial biology–associated genes and upregulation of several genes encoding proinflammatory/fibrotic secreted factors crucial for lung fibroblast activation and IPF progression, including *IGF1*, as the most upregulated, as well as *IGFBP3*, *TGFB2*, *PDGFA*, and *IL1B*. These findings indicate that miR-205-5p drives a transcriptional reprogramming of lung ECs that mirrors the pathological state of IPF-derived lung ECs. The combination of cell cycle arrest and increased secretion of pathogenic factors is a hallmark of cellular senescence (25), a state of stable replicative arrest implicated in IPF pathogenesis (26). Intrigued by these findings, we first confirmed reduced cell cycling by performing an immunofluorescence staining for the proliferation marker Ki67. Quantification of Ki67^+^ cells revealed a significant decrease in proliferating cells upon miR-205-5p overexpression (Figure 4, A and B), confirming our transcriptional data in Figure 3. Next, we evaluated senescence-associated β-galactosidase (SA-β-Gal), an established senescence biomarker, and observed positive staining in lung ECs transfected with miR-205-5p (Figure 4, C and D), confirming that miR-205-5p promotes a phenotypic switch to senescence. Primary cultured IPF lung ECs also exhibited reduced Ki67 protein levels and positive SA-β-Gal (Figure 4, E–H), implicating for the first time to our knowledge a role for senescence in human IPF lung ECs. Importantly, to support that this IPF lung EC senescent phenotype is not an artifact of culturing conditions, we analyzed a publicly available sequencing dataset (27) and confirmed that *CDKN1A*, a key marker of senescence, was upregulated in IPF lung ECs compared with healthy lung ECs (Supplemental Figure 3).

### Overexpression of miR-205-5p in healthy lung ECs induces a pathogenic secretory phenotype that promotes paracrine fibroblast activation.

**Figure 5:**
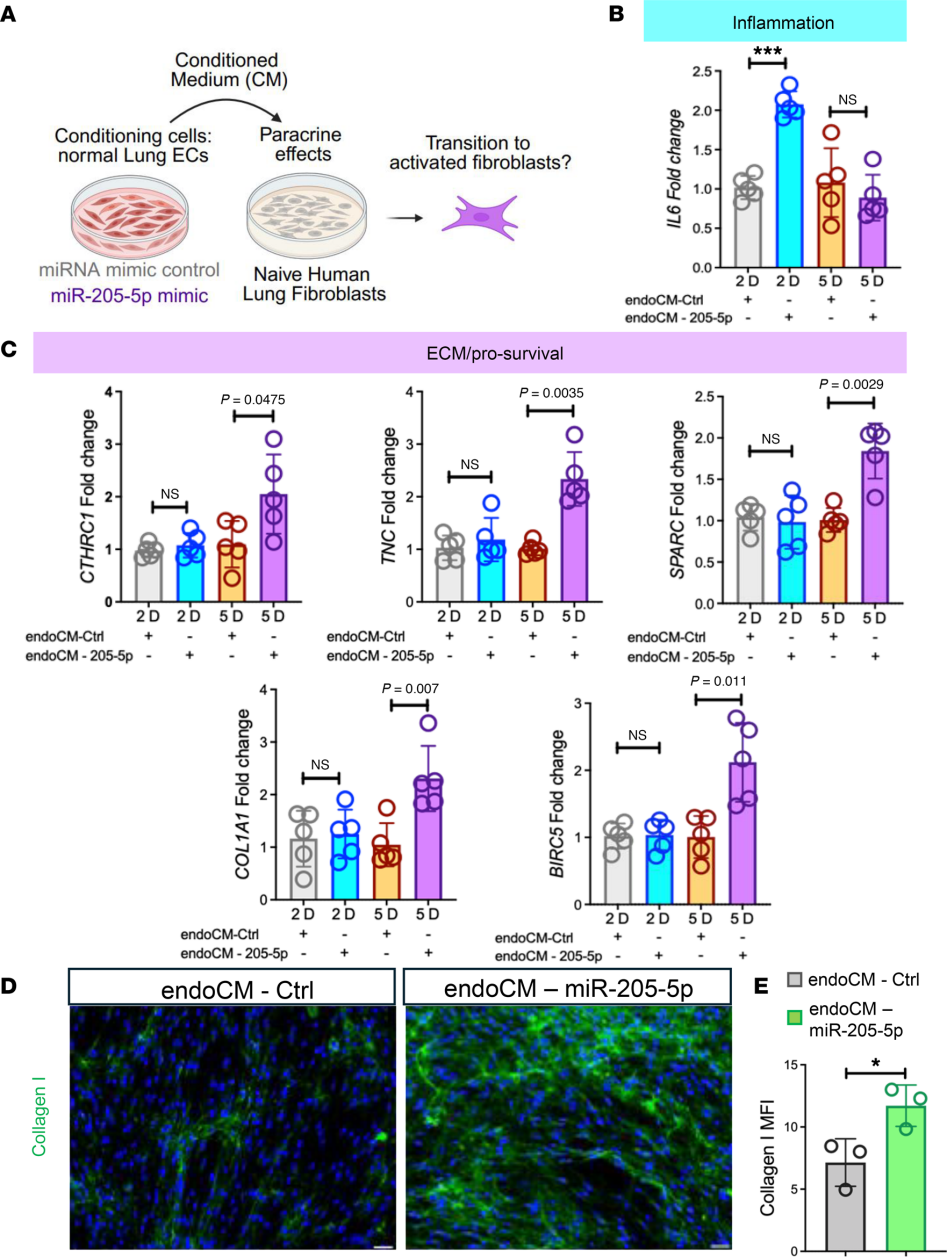
Lung endothelial miR-205-5p regulates paracrine lung fibroblast activation. (**A**) Healthy lung ECs were transfected with a miRNA mimic–negative control or a miR-205-5p mimic. Six hours after transfection, medium was changed to remove transfection reagents. After 3 days, conditioned medium (CM) was collected and applied to recipient healthy human lung fibroblasts for 2 or 5 days (created with a licensed version of BioRender.com). (**B** and **C**) qPCR shows increased expression of proinflammatory *IL6* marker at day 2 and ECM/scar-forming fibroblasts genes at day 5 in healthy human lung fibroblasts exposed to CM from miR-205-5p–overexpressing lung ECs compared with fibroblasts exposed to CM from lung ECs transfected with miRNA mimic–negative control. *N* = 5 independent biological replicates. Data are expressed as mean ± SD, and *P* values were calculated using 1-way ANOVA (followed by Tukey’s post hoc test). (**D**) Representative image of immunostaining for type I collagen in human lung fibroblasts that received CM from control or miR-205-5p–overexpressing lung ECs. (**E**) Quantification of collagen I staining in **D** shows increased collagen deposition in recipient human lung fibroblasts exposed to CM from miR-205-5p–overexpressing lung ECs. *N* = 3 independent biological replicates, each performed in triplicates. Data are expressed as mean ± SD, and *P* values were calculated using Student’s *t* test. *P < 0.05; ***P < 0.001.

Given that miR-205-5p upregulates transcripts encoding secreted profibrotic molecules and induces a senescent switch in lung ECs, we hypothesized that this phenotype results in the secretion of paracrine factors with profibrotic activity on quiescent human lung fibroblasts. To test whether miR-205-5p overexpression in lung ECs modulates fibroblast activation via paracrine communication, we collected conditioned medium from healthy lung ECs transfected with a miR-205-5p mimic or a negative control mimic for 3 days. Human lung fibroblasts were exposed to these conditioned media and harvested after 2 (acute exposure) or 5 (chronic exposure) days, followed by qPCR for a panel of inflammation/fibrosis-related genes (Figure 5A). Upon acute exposure, transcript levels of the inflammatory marker *IL6* were significantly upregulated, while no differences were observed in profibrotic genes (Figure 5B). Intriguingly, following chronic exposure, *IL6* transcripts were downregulated to levels similar to baseline, whereas the prosurvival gene *BIRC5* and a panel of genes associated with scar-forming fibroblasts, including *CTHRC1*, *TNC*, *SPARC*, and *COL1A1*, were significantly upregulated, indicating a shift from an inflammatory to a profibrotic fibroblast phenotype (Figure 5C). To test whether the increase in profibrotic gene expression translated into increased protein production, we performed a collagen I deposition assay and observed significant accumulation of collagen I (Figure 5, D and E). Altogether, these data suggest that endothelial miR-205-5p mediates the secretion of profibrotic molecules that promote the transition of quiescent fibroblasts into scar-forming fibroblast, propagating fibrosis.

### miR-205-5p affects endothelial function potentially by targeting YAP and CDKN1A signaling pathways, and its inhibition in IPF lung ECs mitigates their profibrotic phenotype.

**Figure 6:**
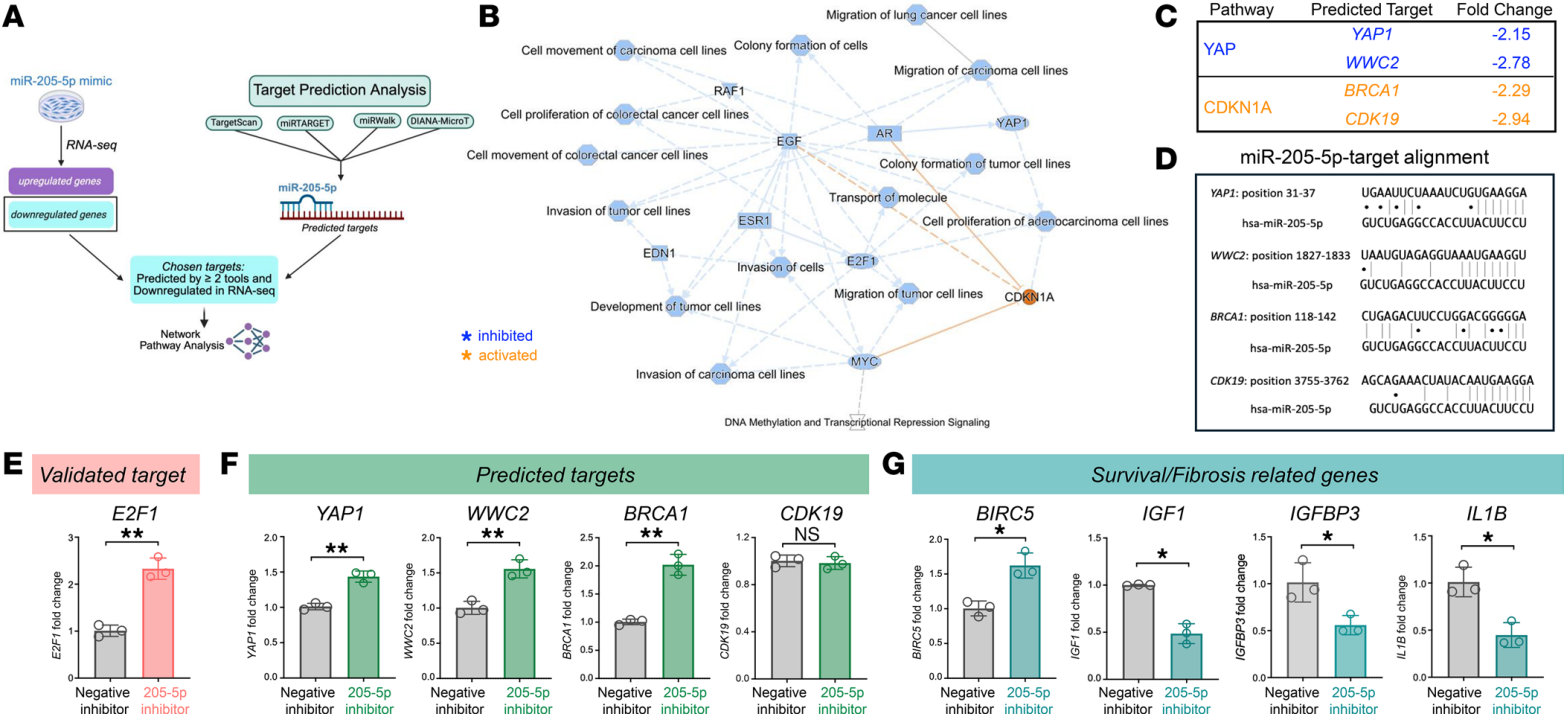
miR-205-5p affects endothelial function potentially by targeting the YAP and CDKN1A cascades, and its inhibition in IPF lung ECs partially mitigates their profibrotic phenotype. (**A**) Schematic depicting our target prediction analysis workflow (created with a licensed version of BioRender.com). (**B**) Network pathway analysis (IPA) of genes predicted to be targets of miR-205-5p in our study. (**C**) Selected predicted target genes in YAP and CDKN1A cascades. (**D**) Predicted miR:target alignment region. Lines indicate Watson-Crick base pairs; dots indicate G:U wobble base pairs. (**E**–**G**) qPCR showing changes in miR-205-5p predicted target genes and genes associated to endothelial dysfunction in IPF lung ECs transfected with a miR-205-5p inhibitor. Inhibition of miR-205-5p leads to the upregulation of the predicted target genes *YAP1*, *WWC2*, and *BRCA1*; upregulation of the survival gene *BIRC5*; and downregulation of profibrotic genes *IGF1* and *IL1B*. The predicted target gene *CDK19* remains unchanged. *N* = 3 independent biological replicates. Data are expressed as mean ± SD, and *P* values were calculated using Student’s *t* test. *P < 0.05; **P < 0.001.

**Figure 7:**
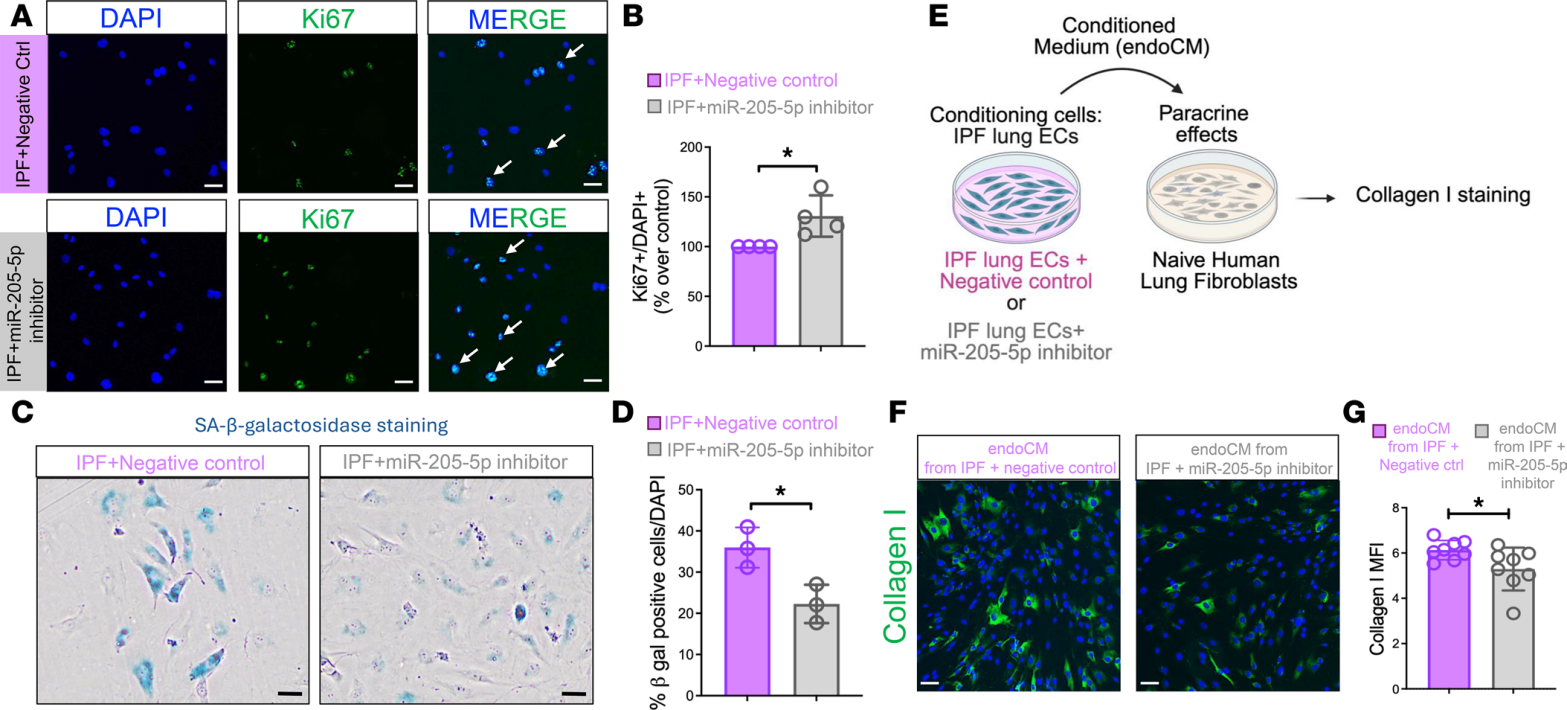
miR-205-5p inhibition attenuates IPF lung EC senescence and limits paracrine fibroblast activation. (**A**) Representative immunofluorescence images for Ki67 and DAPI in IPF lung ECs transfected with a negative miRNA inhibitor or miR-205-5p inhibitor. Arrows indicate Ki67^+^ nuclei. Scale bar: 20 μm. (**B**) Quantification of Ki67^+^ nuclei (normalized to DAPI) reveals a significant increase in miR-205-5p–inhibited IPF lung ECs versus control-transfected IPF cells. Data are shown as mean ± SD of *n* = 4 independent biological replicates (average of 3–5 images per replicate) and expressed as percentage over control. *P* values were calculated using Student’s *t* test. (**C**) Representative SA-β-gal staining images showing attenuated staining in miR-205-5p–inhibited IPF lung ECs. Scale bar: 20 μm. (**D**) Quantification of SA-β-gal–positive cells (over total DAPI-positive cells) showing reduction of SA-β-gal–positive cells in miR-205-5p–inhibited IPF lung ECs. Data are shown as mean ± SD of *n* = 3 independent biological replicates (average of 3–5 images per replicate), and *P* values were calculated using Student’s *t* test. (**E**) IPF lung ECs were transfected with a miRNA inhibitor negative control or a miR-205-5p inhibitor. Six hours after transfection, medium was changed to remove transfection reagents. After 3 days, conditioned medium (CM) was collected and applied to recipient quiescent human lung fibroblasts for 5 days (schematics created with a licensed version of BioRender.com). (**F**) Representative images of immunostaining for type I collagen in human lung fibroblasts that received CM from control IPF lung ECs or from IPF lung ECs after miR-205-5p inhibition. (**G**) Quantification of collagen I staining in **E** shows decreased collagen deposition in recipient human lung fibroblasts exposed to CM from IPF lung ECs upon inhibition of miR-205-5p. *N* = 8 independent biological replicates, each performed in triplicates. Scale bar: 20 μm. Data are expressed as mean ± SD, and *P* values were calculated using Student’s *t* test. *P < 0.05.

Growing evidence highlights the therapeutic potential of miRNAs as a promising new class of targeted interventions. Because miRNAs can regulate several gene expression programs (28), identifying their targets in a specific biological context is crucial for understanding their function. Since our RNA-seq analysis had already identified genes downregulated by miR-205-5p overexpression (Figure 3), we first used miRTARGET, which enables enrichment analysis based on predicted miRNA-target interactions, to analyze the list of downregulated genes. This unbiased analysis confirmed miR-205-5p as the most enriched miRNA, supporting the validity of our approach (Supplemental Figure 4A). To further elucidate the mechanisms by which miR-205-5p contributes to endothelial dysfunction and fibrosis, and to restrict the analysis to genes relevant to lung endothelial biology, we performed gene target prediction and intersected it with the significantly downregulated genes in our RNA-seq. To minimize false-positive target assignments, target prediction analysis was performed with 4 independent target prediction platforms (TargetScan, miRTARGET, miRWalk, DIANA MicroT), and results were integrated with our RNA-seq. Thus, in downstream analyses, we only included genes that were predicted to be targets by at least 2 independent platforms and were significantly downregulated by miR-205-5p in lung ECs (Figure 6A). To gain mechanistic insight into the pathways regulated by miR-205-5p in the lung endothelium, we employed a network-based pathway analysis on the predicted targets (complete target list in Supplemental Table 1). The analysis considered how predicted miR-205-5p targets interact with other genes within network pathways, providing a broad understanding of the potential impact of miR-205-5p in the lung endothelium. Intriguingly, among the regulated pathways, the YAP cascade, a key component of the Hippo signaling involved in fibrosis, was predicted to be inhibited, whereas the CDKN1A pathway, associated to cellular senescence, was predicted to be activated (Figure 6B). These findings suggest that both pathways may contribute to the profibrotic phenotype induced by miR-205-5p in lung ECs. Within these pathways, we identified *YAP1* and *WWC2* as predicted targets of the YAP cascade and *BRCA1* and *CDK19* as predicted targets of the CDKN1A cascade (Figure 6C, target alignment of miR-205-5p for predicted targets shown in Figure 6D). To extend these in silico predictions to the context of human IPF lung ECs, we performed a miR-205-5p inhibition assay and assessed the expression of the 4 predicted target genes. Efficient miR-205-5p inhibition was confirmed using the validated target *E2F1* (29–31) as an internal control, which showed significant upregulation following miR-205-5p inhibition (Figure 6E). Notably, *YAP1*, *WWC2*, and *BRCA1* were significantly upregulated upon miR-205-5p inhibition, supporting their potential as direct targets, whereas *CDK19* expression remained unchanged (Figure 6F). Importantly, inhibition of miR-205-5p in IPF lung ECs resulted in upregulation of *BIRC5*, which we found downregulated in cultured IPF lung ECs (Figure 3H), suggesting improved endothelial survival, and in downregulation of *IGF1* (the most upregulated profibrotic gene in our analysis of IPF lung ECs), *IGFBP3,* and *IL1B* transcripts, key mediators of fibrotic remodeling in the lung and components of the SASP that were all upregulated in cultured IPF lung ECs (Figure 6G). While the expression of the profibrotic genes *TGFB2* and *PDGFA*, as well as the endothelial identity gene *NOS3*, was not changed by the inhibition of miR-205-5p in IPF lung ECs (Supplemental Figure 4B), overall, these data suggest that inhibition of miR-205-5p in IPF lung ECs may attenuate their profibrotic phenotype. To test this hypothesis, we transfected IPF lung ECs with a negative inhibitor control or a miR-205-5p inhibitor and demonstrated increased cell proliferation, as assessed as Ki67^+^ cells (Figure 7, A–C), and attenuated SA-β-Gal expression (Figure 7, D and E) upon miR-205-5p inhibition in IPF cells, compared with control IPF cells transfected with a negative inhibitor control. Prompted by these data, we then tested whether miR-205-5p inhibition would mitigate the profibrotic activity of the conditioned medium generated by IPF lung ECs. Intriguingly, the conditioned medium generated by IPF lung ECs transfected with a miR-205-5p inhibitor exhibited reduced paracrine profibrotic activity, as demonstrated by reduced collagen I production by human lung fibroblasts exposed to the conditioned medium generated by miR-205-5p–inhibited IPF lung ECs compared with the control-transfected IPF lung ECs (Figure 7, F and G). Altogether, these findings suggest that miR-205-5p impairs endothelial function potentially via the YAP and CDKN1A cascades, and that its inhibition attenuates the profibrotic endothelial phenotype of IPF lung ECs, highlighting its potential as a therapeutic target.

### Inhibition of miR-205-5p attenuates lung fibrosis progression in aged mice challenged with bleomycin.

**Figure 8:**
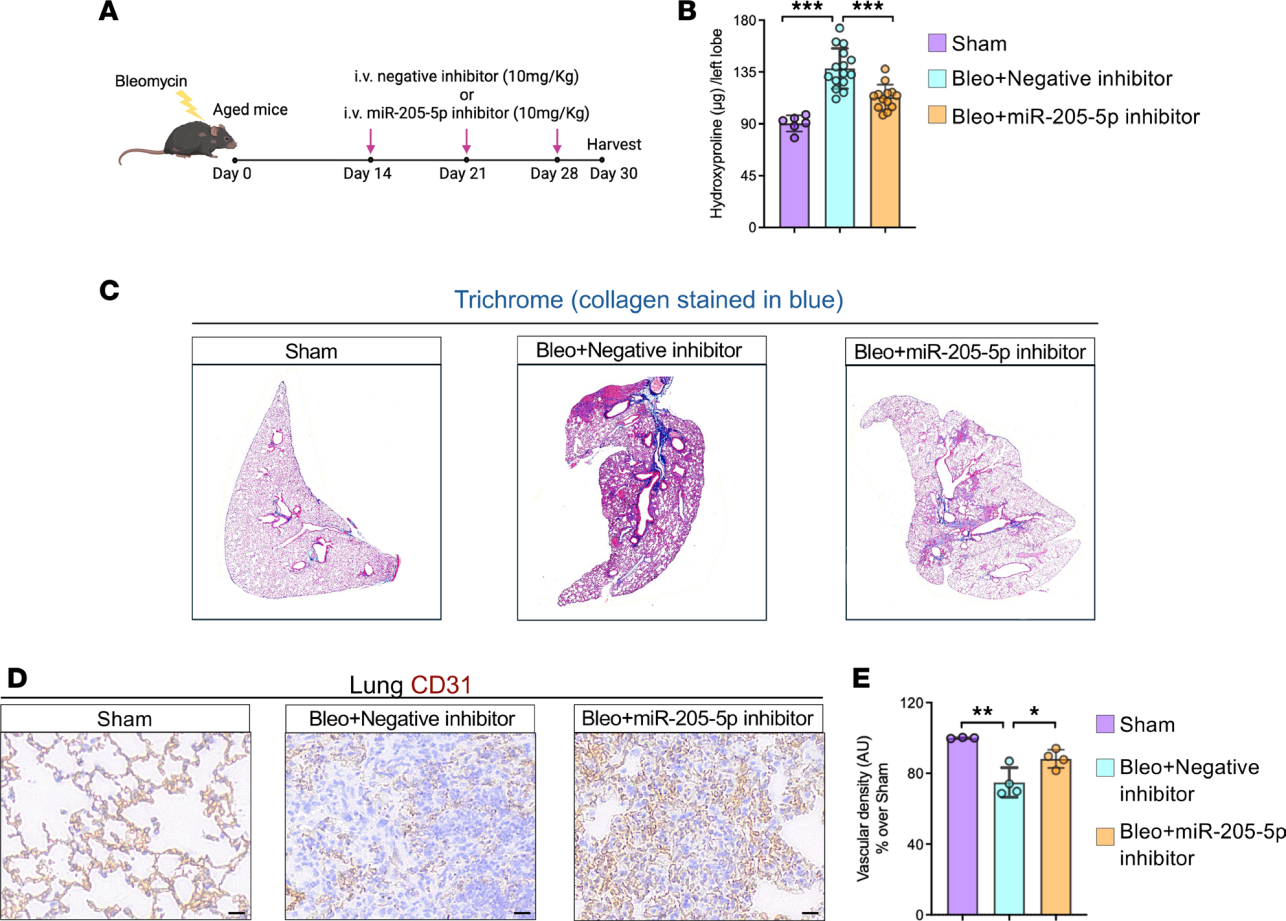
Inhibition of miR-205-5p mitigates fibrosis progression in aged mice challenged with bleomycin. (**A**) Schematic illustration of the experimental design for the miR-205-5p inhibitor administration, starting at day 14 after bleomycin injury to assess effects on progression of fibrosis (created with a licensed version of BioRender.com). (**B**) Hydroxyproline assay quantifying total collagen content in lungs from sham-operated mice, mice administered bleomycin + negative inhibitor control, and mice administered bleomycin + miR-205-5p inhibitor at day 30 after injury. Values are summarized as mean ± SD, and *P* values were generated using 1-way ANOVA with Tukey’s post hoc test for comparison. Sham (*n* = 11), bleomycin + negative inhibitor control (*n* = 12), and bleomycin + miR-205-5p inhibitor (*n* = 13). (**C**) Representative Masson’s trichrome staining in lung sections from sham, bleomycin-injured control–, and bleomycin-injured miR-205-5p inhibitor–treated mice at day 30, demonstrating attenuated collagen (stained in blue) deposition with miR-205-5p inhibition. (**D** and **E**) Immunohistochemistry of mouse lung sections and relative quantification revealed reduced vessel density (CD31-positive) in bleomycin-injured lungs. The vascular network of the lungs of mice treated with miR-205-5p inhibitor was improved compared with that of negative inhibitor–treated bleomycin-injured mice. Values are summarized as mean ± SD, and *P* values were generated using 1-way ANOVA with Tukey’s post hoc test for comparison. Each dot represents an individual mouse, and for each mouse an average of 5–10 fields of view have been used for the analysis. Sham (*n* = 3), bleomycin + negative inhibitor control (*n* = 4), and bleomycin + miR-205-5p inhibitor (*n* = 4).

Given that miR-205-5p inhibition in IPF lung ECs attenuated their profibrotic phenotype, and because previous studies, including ours (6–9), showed that mitigating endothelial dysfunction reduces fibrosis progression, our next goal was to assess the therapeutic potential of miR-205-5p inhibition in bleomycin-induced chronic fibrosis in aged mice. The miR-205-5p sequence is conserved across vertebrates, including within seed regions mediating target recognition, supporting the expectation that the effects observed in human cells would be preserved in mice. To test the effect of in vivo miR-205-5p inhibition, we delivered a specific short oligonucleotide miR-205-5p inhibitor intravenously. Successful uptake by the pulmonary vasculature was confirmed by injecting a fluorescently labeled (FAM) inhibitor via retro-orbital injection followed by microscopy imaging, which detected the FAM-labeled miR-205-5p inhibitor in the pulmonary vasculature, detected via PECAM1 staining (Supplemental Figure 5). For subsequent experiments, mice were treated with miR-205-5p inhibitor, or a negative inhibitor control, at a concentration of 10 mg/kg (based on published evidence using the same miRNA inhibitors in preclinical mouse studies (17) via retro-orbital injection, once per week, beginning at day 14 after bleomycin delivery, after the initial inflammatory phase, based on previous findings from our group and others (32, 33), and lungs were harvested at day 30, time point associated with chronic fibrosis as previously demonstrated (7, 9) (Figure 8A). To assess the effect of miR-205-5p inhibition on bleomycin-induced lung fibrosis, we measured collagen content by hydroxyproline assay, which revealed significantly lower hydroxyproline levels in mice treated with the miR-205-5p inhibitor compared with the negative control inhibitor group (Figure 8B). Consistently, Masson’s trichrome staining revealed a reduction in collagen deposition in mice treated with miR-205-5p inhibitor (Figure 8C). Furthermore, following bleomycin injury, regions of dense cellularity in control mice treated with the negative miRNA inhibitor exhibited reduced vessel density, consistent with our previous observations (9, 12). In contrast, miR-205-5p inhibition preserved vessel density in these regions (Figure 8, D and E), aligning with our previous findings showing that preservation of vascular integrity is associated with mitigation of bleomycin-induced lung fibrosis in aged mice (12). Taken together, our findings indicate that miR-205-5p inhibition attenuates lung fibrosis progression in vivo, highlighting its potential as therapeutic avenue in lung scarring.

## Discussion

Research on the contribution of vascular alterations to the pathogenesis and progression of IPF has advanced rapidly, yet significant knowledge gaps persist. Emerging evidence, including our own, has implicated dysfunctional endothelial transcriptional programs in progressive lung fibrosis (5, 7, 9–11). However, the exact mechanisms driving these transcriptional alterations remain poorly understood. miRNAs are a large family of small noncoding RNAs that have emerged as key regulators of gene expression (15). Studies have demonstrated that miRNAs participate in the regulation of multiple cellular processes and that alterations of miRNA expression are associated with the onset and progression of a plethora of human diseases (34), including IPF (35). miRNA research in IPF has primarily focused on fibroblasts, epithelial cells, and macrophages. Recent research has shown that in macrophages, miR-33 is increased and regulates immunometabolic responses in IPF. In lung epithelial cells, studies have identified miR-21, let7-d, and miR-26a as inducers of epithelial-mesenchymal transition and profibrotic epithelial phenotypes (36–38). In fibroblasts, miR-21, miR-29, and let-7 have been identified as important miRNAs regulating fibroblast activation (39). Notably, the specific role of miRNA in regulating lung EC gene expression during IPF remains largely unexplored.

This study is the first to our knowledge to profile miRNAs in fibrotic lung ECs, which are increasingly recognized as key contributors to IPF progression. Our initial miRNA screening revealed elevated levels of miR-205-5p and miR-34a-5p in ECs freshly isolated from aged mice with persistent lung fibrosis. Further analysis of lung ECs isolated from healthy and IPF lungs confirmed the overexpression of miR-205-5p, but not of miR-34a-5p, despite the fact that the miR-34 family has been implicated as a promoting factor of hepatic fibrosis; this thus indicates a conserved role for miR-205-5p across both murine and human fibrotic remodeling of the lung.

The role miR-205-5p has been investigated for its functions in development and cancer, where it is aberrantly expressed and may exert pro- or antitumorigenic roles depending on the cellular context and target genes (29, 31, 40, 41). In lung cancer, miR-205-5p promotes tumor progression, and its serum levels are significantly higher in patients compared with individuals acting as controls and correlate with patient’s clinical stage (41). In addition, miR-205-5p has several important effects in vascular EC function, including inhibition of angiogenesis and tube formation (42, 43). Comparatively little attention has been focused on the role of miR-205-5p in lung injury, repair, and fibrosis. One recent study showed reduced miR-205-5p levels in lungs of mice with silica-induced pulmonary fibrosis and suggested that miR-205-5p could play a protective role against lung fibrosis (44). In contrast, miR-205 was found upregulated in lung tissue of patients with IPF compared with that of individuals acting as controls (45), suggesting that this miRNA is implicated in progressive lung fibrosis and that further work is needed to elucidate its overall contribution to the disease. In this work, we demonstrate that miR-205-5p induces transcriptional alterations in lung ECs, including downregulation of genes associated with cell cycle progression and endothelial identity/function, while simultaneously upregulating genes encoding secreted mediator of fibrosis and component of the SASP, a transcriptional profile that mirrors the phenotype of cultured IPF lung ECs. Further analyses confirmed that miR-205-5p induces cell cycle arrest and promotes a prosenescent endothelial phenotype, recapitulating the behavior of lung ECs isolated from IPF lungs. Cellular senescence markers have been detected in IPF lung tissue, with established senescence biomarkers such as SA- β -galactosidase observed in both fibroblasts and epithelial cells (26). However, the contribution of endothelial senescence has remained largely unexplored. Our findings provide the first evidence to our knowledge of senescence in the lung endothelium of human fibrotic lungs and identify miR-205-5p as a key inducer of this pathogenic phenotype.

Cell-cell interactions are essential for coordinated responses during homeostasis and injury responses. We and others have demonstrated that lung endothelial dysfunction results in a profibrotic change of the endothelial secretome (5, 7, 9–11), and senescent cells are known to secrete a broad repertoire of SASP factors that influence neighboring cells via paracrine signaling, many of which are also profibrotic mediators (26). Consistent with these observations, we found that conditioned medium from miR-205-5p–overexpressing lung ECs promotes the transition of quiescent fibroblasts into scar-forming fibroblasts, indicating a potentially novel connection between lung endothelial miR-205-5p and profibrotic alterations of the endothelial secretome. Intriguingly, these effects were temporally regulated, as the 2-day acute exposure induced upregulation of the inflammatory marker IL-6, whereas the 5-day chronic exposure upregulated the expression of markers of scar-forming fibroblasts. This sequential induction of inflammatory and fibrotic fibroblasts states is in agreement with recent findings demonstrating that during both mouse and human lung fibrosis, alveolar fibroblasts undergo a sequential differentiation into inflammatory and then fibrotic fibroblasts (27). These results, together with the spatial distribution of miR-205-5p in vessels adjacent to nascent fibroblast foci in IPF lung tissue, suggest that EC-fibroblast crosstalk induced by endothelial miR-205-5p contributes to the progression of pathogenic fibroblasts in vivo. Notably, a recent study showed that *MIR205HG*, a long noncoding RNA and host gene for miR-205, is highly expressed in aberrant basal cells and contributes to IPF pathogenesis via upregulation of IL33 (46). Interestingly, the authors showed that miR-205 had no profibrotic effects in alveolar organoids, consistent with reports that *MIR205HG* functions independently of its hosted miR-205 in other contexts (47).

Our target prediction analysis of genes downregulated following miR-205-5p overexpression in lung ECs integrated with pathways analysis highlighted the YAP and CDKN1A signaling cascades as potential contributors to the miR-205-5p–induced profibrotic endothelial phenotype. Both pathways have been implicated in fibrotic remodeling of the lungs, although their specific contribution within the lung endothelium remains poorly defined. The significant overexpression of predicted target genes *YAP1*, *WWC2* (associated with the YAP pathway), and *BRCA1* (associated with the CDKN1A pathway) following inhibition of miR-205-5p in IPF lung ECs supported our findings. Although we did not observe upregulation of *CDK19,* we cannot exclude that miR-205-5p regulates its expression at protein level in IPF lung ECs. Further mechanistic studies are warranted to confirm direct targets and determine their causative role in the profibrotic endothelial phenotype induced by miR-205-5p. Given our previous findings demonstrating that activation of the YAP pathway drives fibrotic endothelial remodeling (11), defining whether and how miR-205-5p connects with this pathway will be an important avenue of future investigations. Nevertheless, our data demonstrate that inhibition of miR-205-5p in IPF lung ECs induces the expression of *BIRC5*, potentially improving endothelial survival, while concurrently suppressing key SASP and profibrotic mediators *IGF1*, *IGFBP3*, and *IL1B*, which are highly upregulated in IPF lung ECs and implicated in fibrotic lung remodeling. Although other profibrotic genes, including *TGFB2* and *PDGFA*, remained unchanged, these results collectively support the concept that miR-205-5p inhibition could mitigate endothelial dysfunction and profibrotic signaling in lung fibrosis progression.

Over recent years, interest in miRNAs as therapeutic targets has grown, driven by promising results from preclinical studies in several diseases, including IPF. Notably, Chioccioli and colleagues developed a miRNA mimic to miRNA-29b that demonstrated antifibrotic activities both in vitro and in vivo, highlighting the potential for novel miRNA-based therapeutic approaches (48). Our in vitro data showed that inhibition of miR-205-5p in IPF lung ECs promotes cell proliferation and reduces the number of SA-β-gal–positive cells, demonstrating that this strategy is effective in attenuating features of IPF endothelial dysfunction and senescence. Notably, these findings align with literature showing that other miRNAs (i.e., miR-195) can reduce the SA-β-gal expression of old mesenchymal stem cells (49). Further experiments showed that inhibition of miR-205-5p in IPF lung ECs mitigated their profibrotic paracrine effects on human fibroblasts, indicating that this approach could effectively halt lung fibroblast activation and fibrosis progression. This hypothesis was validated by in vivo findings showing that miR-205-5p inhibition protects the vascular network and mitigates fibrosis progression in bleomycin-induced chronic fibrosis in aged mice, highlighting the potential for future development of EC-targeted miRNA-based therapeutic strategies. Although no systemic miRNA-based therapy has yet achieved regulatory approval, largely due to immune-mediated adverse events and off-targets effects (50), the growing development of nanotherapeutics capable of precisely targeting specific lung cell types, including ECs, as recently demonstrated by our group and others (32, 51), opens new avenues for EC-specific miR-205-5p inhibition as a therapeutic strategy for fibrotic lung disease potentially minimizing immune-mediated adverse events and off-target extrapulmonary effects.

This study has 2 main limitations. First, we did not perform a small RNA-seq as initial screen, which could have revealed additional relevant miRNAs. However, our targeted approach focusing on miRNAs involved in fibrosis, inflammation, and endothelial biology successfully identified a miRNA with a key role in lung fibrosis. Second, we did not comprehensively study the secretome induced by miR-205-5p and its relationship with the miR-205-5p targetome in lung ECs. Therefore, future work will focus on directly validating the lung endothelial miR-205-5p targetome and assessing the sufficiency of candidate target genes in inducing the altered endothelial secretome downstream of miR-205-5p and identifying secreted factors and their potential interaction with cells in the alveolar niche beyond fibroblasts. Additional future directions of this work include identifying cues driving endothelial miR-205-5p upregulation and profibrotic phenotype alterations. Given that miR-205 is upregulated by shear stress in models of flow-induced atherosclerosis (52), and fibrotic lungs exhibit altered mechanical environment, mechanical changes likely play a role in driving its endothelial expression.

In conclusion, our in vitro and in vivo findings identify endothelial miR-205-5p as a pivotal mediator of endothelial dysfunction and driver of lung fibrosis progression. Therefore, targeting endothelial miR-205-5p, or its downstream effector pathways, represents a promising therapeutic opportunity to mitigate fibrotic remodeling in the lung.

## Methods

### Sex as a biological variable.

Our study examined male and female mice, and similar findings are reported for both sexes. Owing to limited number of patient samples used in this study, statistical adjustment for sex or gender analysis was not performed. In this study, we used lung tissue from 4 healthy donors (3 males and 1 female) and 7 patients with IPF (5 males and 2 females).

### Patient samples.

Lung tissue from patients with IPF was obtained from explanted lungs obtained at the time of transplantation. All patients provided written informed consent, and the study was approved by the Loyola University Chicago Institutional Review Board (#216973). Diagnoses of patients with IPF were established by clinical, radiological, and pathological criteria and confirmed by multidisciplinary consensus conference. Healthy control lungs were obtained from deceased donors whose lungs were deemed unsuitable for transplant and were provided by Gift of Hope with consent from family for tissue to be used for research purposes. No compensation was provided to participants or family for either IPF patient samples or healthy control lungs.

### Cell culture.

Lung ECs from patients diagnosed with IPF as well as nonfibrotic healthy individuals acting as controls were generated in-house using the following protocol. Whole lungs were mechanically and chemically digested. At least 5 grams of whole lung tissue was minced with a razor blade and transferred to 50 mL conical tube with 3.75 mL/g of digestion buffer with 1.5 mg/mL collagenase type IV (Worthington Biochemical Corporation), 1 mg/mL collagenase type I (Worthington Biochemical Corporation), and 100 U/mL of DNase I (Roche). Tissue was incubated at 37°C with rotation for 1 hour. Digested tissue was filtered through sterile gauze, then 100 μm cell strainer, and finally a 30 μm cell strainer to obtain a single-cell suspension. Whole cell suspension was pelleted, and red blood cells were lysed using 1 mL red blood cell lysis buffer per gram of starting lung tissue. Red blood cell lysis buffer was neutralized with 3 volumes of PBS, and cells were strained through a 30 μm cell strainer. Subsequently, the whole-cell suspension was pelleted and finally resuspended in Promocell Endothelial Cell Growth Medium MV2 (Promocell) complete media with 1% Pen/Strep. Whole cell suspension was seeded in collagen-coated flasks (PureCol type I bovine collagen solution, Advanced BioMatrix). When cells were confluent, CD31-dynabeads were used to purify lung ECs (Thermo Fisher Scientific) that were cultured with Promocell Endothelial Cell Growth Medium MV2 (Promocell). Human lung fibroblasts were purchased from Angioproteomie and cultured in DMEM/F12 with 10% FBS and 1% Pen/Strep.

### Mouse studies.

All animal studies were performed under protocols approved by the Mayo Clinic Institutional Animal Care and Use Committee and Loyola University Chicago’s Institutional Animal Care and Use Committee and conform to the Animal Research: Reporting of In Vivo Experiments guidelines. Col1α1-GFP transgenic mice (FVB strain) were provided by Derek Radisky (Mayo Clinic). C57BL/6J mice were purchased from The Jackson Laboratory or obtained via the National Institute on Aging. Mice had access to food and water ad libitum and were on a 12/12-light/dark cycle, with ambient temperature 77°F–78°F and humidity 46%–49%.

### Mouse model of bleomycin-induced lung fibrosis.

Young (2-month-old) and aged (18-month-old) mice, Col1a1 GFP transgenic mice (FVB strain), both female and male, were anesthetized with a solution of ketamine (100 mg/kg) and xylazine (10 mg/kg), injected intraperitoneally. Bleomycin 1.2 U/kg (APP Pharmaceutical LCC) or PBS were intratracheally delivered as described in our previous work (53).

For the repetitive injury model, pulmonary fibrosis was induced in young (2-month-old) C57BL/6J mice, both male and female, by 6 intratracheal instillation of bleomycin (0.8 U/kg) given biweekly while mice were under isoflurane anesthesia. Mice were euthanized 3 months after the last bleomycin instillation. Mice receiving a single dose of bleomycin or PBS were used as controls. To assess the effect of miR-205-5p inhibition in mitigation of chronic fibrosis progression, we challenged 18-month-old C57BL/6J male and female mice with a single dose of bleomycin, administered as described above. miRCURY Power LNA miRNA inhibitors (negative control, GeneGlobe, YCI0202032 - FZA, or against miR-205-5p GeneGlobe, YCI0201821 - FZB, Qiagen - test injections with 5’FAM-labeling) were administered starting on day 14 after bleomycin (1 intravenous retro-orbital injection, weekly, at concentration of 10 mg/kg, based on manufacturer’s recommendations and published evidence; ref. 54, 55), and lungs were harvested 30 days after injury, as in our previous work (31). At least 5 mice were instilled for each experimental condition on the basis of a power analysis to detect significant differences in fibrosis (56), and numbers indicated in the figure legends.

### Masson’s trichrome staining.

Right lobes were inflated and submerged with 10% neutral buffered formalin for 24 hours and then transferred to 70% EtOH before being paraffin embedded. Masson’s trichrome staining was performed by American Histolabs, or by the Research Histology Core at the University of Illinois Chicago, using 5 μm lung sections.

### FACS.

Mice were anesthetized with a solution of ketamine (100 mg/kg) and xylazine (10 mg/kg), injected intraperitoneally, and perfused via the left ventricle with cold PBS 30 days after bleomycin or PBS delivery. The lungs were immediately harvested and minced with a razor blade in a 100 mm petri dish in a cold DMEM medium containing 0.2 mg/mL Liberase DL and 100 U/mL DNase I (Roche). The mixture was transferred into 15 mL tubes and incubated at 37°C for 35 minutes under continuous rotation to allow enzymatic digestion. Digestion was inactivated with DMEM medium containing 10% fetal bovine serum, the cell suspension was passed through a 40 μm cell strainer (Fisher) to remove debris. Cells were then centrifuged (500*g*, 10 minutes, 4°C) and resuspended in 3 mL red blood cell lysis buffer (Biolegend) for 90 seconds to remove the remaining red blood cells and diluted in 9 mL PBS after incubation. Cells were then centrifuged (500*g*, 10 minutes, 4°C) and resuspended in 0.2 mL of FACS buffer (1% BSA, 0.5 mM EDTA pH 7.4 in PBS). For sorting, the single-cell suspension was then incubated with anti-CD45:PerCp-Cy5.5 (BioLegend, 103132, RRID:AB_893340, 1:200 dilution), anti-CD31:PE (BioLegend, 102407, RRID:AB_312902, 1:200 dilution), anti-EpCAM:APC (BioLegend, 118213, RRID:AB_1134105, 1:200 dilution) antibodies, and DAPI (D3571, Thermo Fisher Scientific, 1:1,000 dilution) for 30 minutes on ice. After incubation, cells were washed with ice-cold FACS buffer and resuspended in 1 mL of FACS buffer. FACS sorting was conducted using a BD FACS Aria II (BD Biosciences). To isolate CD45^–^, EpCAM^–^, GFP^–^, CD31^+^ populations the following strategies was used: debris exclusion (FSC-A by SSC-A), doublet exclusion (SSC-W by SSC-H and FSC-W by FSC-H), dead cell exclusion (DAPI by PE), CD45-positive cell exclusion (PerCP-Cy5.5 by GFP), EpCAM- and GFP-positive cells exclusion (APC by GFP), and isolation of CD31-positive cells (APC by CD31) as previously described (7).

### RNA isolation and miRNA screening.

Total mRNA was isolated from FACS-sorted CD31^+^ ECs isolated from mouse lungs using the RNeasy micro kit (Qiagen), followed by Nanodrop concentration and purity analysis. cDNA was synthesized with SuperScript VILO (Thermo Fisher Scientific); the miRCURY LNA miRNA Focus mouse PCR Panel was used to screen 84 miRNAs. Data represent fold changes of each miRNA relative to the aged sham mice and normalized to the global mean of miR-191-5p, miR-103a-3p, and miR-16-5p; stably expressed miRNAs used as reference miRNAs. Fold changes were then converted to *z* scores. UniSp2, UniSp4, UniSp5, and cel-miR-39-3p RNA were used as Spike-in internal controls.

### Immunofluorescence.

Human lung fibroblasts were seeded on coverslips and then treated for 5 days with conditioned medium generated from (a) healthy lung ECs transfected with a negative control mimic or a miR-205-5p mimic and (b) IPF lung ECs, transfected with a negative control inhibitor or a miR-205-5p inhibitor. In experiments involving lung ECs, cells were seeded and grown on coverslips. Cells were then fixed with 10% formalin for 10 minutes at room temperature and washed 3 times with ice-cold PBS. Cells were then permeabilized with 0.1% Triton X-100 for 5 minutes. Following 3 washes with PBS, cells were incubated with blocking buffer (5% goat serum, 2% BSA) for 1 hour and then stained with collagen I at 1:200 dilution (72026, Cell Signaling Technology) for fibroblasts and Pecam1 at 1:200 dilution (3528, Cell Signaling Technology) for ECs overnight at 4°C. Next day, cells were washed 3 times with PBS and incubated with secondary antibodies (A11001 and A11008, Thermo Fisher Scientific) at 1:1,000 and containing DAPI stain (1 mg/mL, dilution 1:1,000) for 1 hour. Following 3 washes with PBS, coverslips were mounted onto slides using Aqua Poly mounting medium (Polysciences Inc.) and then imaged using a fluorescent microscope equipped with a camera (Olympus CKX53).

### Immunohistochemistry.

FFPE mouse lungs were cut in serial sections (7 μm). The FFPE sections were deparaffinized using a standard protocol of xylene and alcohol gradients. Sections were then blocked, first with BLOXALL endogenous peroxide blocker (SP-6000-100, Vector Laboratories) and then with 5% goat serum and 2% BSA (Sigma-Aldrich). Staining was performed using the VECTASTAIN Elite ABC HRP kit (PK-6200, Vector Laboratories) and anti-CD31 rat antibody (550274, clone MEC 13.3, BD Biosciences, 1:200 dilution), detection was performed with impact DAB (Vector Laboratories), and sections were counterstained with hematoxylin. Slides were then dehydrated using a standard protocol and mounted on a coverslip using DPX mounting media (Sigma-Aldrich) (20). Quantification of histochemical staining was performed using the Colour Deconvolution 2 ImageJ plugin on FIJI (NIH) (57). Images of stained tissue sections were acquired using identical microscope settings, and color separation was performed using Colour Deconvolution 2 plugin using DAB specific stain vector. Background signal was subtracted by measuring unstained areas, and thresholds were set to minimize noise. The intensity of staining was measured as integrated density Data were collected from 4–8 fields of view per sample and averaged to obtain a representative value.

### miRNA scope.

Paraffin lung tissue blocks were sectioned at 5 μm thickness. Sections were placed on silanized (3-aminopropyltriethoxysilane) glass slides and incubated in 10% neutral buffered formalin overnight. Slides were then washed twice in 100% ethanol, treated with 3% H_2_O_2_ in ethanol for 10 minutes at room temperature, and incubated at 110°C for 15 minutes in Co-Detection Target Retrieval Reagent (323165, Advanced Cell Diagnostics [ACD]). Following cooling to room temperature, slides were rinsed twice in 100% ethanol, dried for 5 minutes at 60°C, and marked with an ImmEdge Hydrophobic Barrier pen (H-4000, Vector Laboratories). Slides were treated with RNAscope Protease Plus (322381, ACD) for 30 minutes at 40°C and rinsed twice in distilled water. Subsequently, slides were processed for in situ hybridization with the miRNAscope HD (Red) Assay kit (324510, ACD) for human miR-205-5p (728541). After detection of miRNAscope probes using FAST Red chromogen, slides were blocked in codetection blocker (323180, ACD) and incubated overnight with Ulex lectin (CF640R/Far Red, 29112, Biotium) to stain human vasculature and primary antibody against ACTA2 (VIVID520/FITC, 7523, Tocris; dilution 1:100) in CoDetection Antibody Diluent (323180, ACD). The following day, the slides were rinsed and mounted in Vectashield VIBRANCE Plus DAPI (catalog no. H-1800, Vector Laboratories). Digitizing was performed at the Center for Advanced Microscopy at Northwestern University. Image acquisition was performed using the TissueFAXS System (TissueGnostic). Tissue sections were outlined, and integrated density was calculated using FIJI (NIH). as previously described (46).

### Transfection of negative control and miR-205-5p mimics.

Healthy lung ECs were plated at 40,000 cells per well of a 12-well plate in 1 mL of complete media Promocell Endothelial Cell Growth Medium MV2 1% Pen/Strep (Promocell). The next day cells were transfected with mirVana-negative control mimic or miR-205-5p mimic, using Lipofectamine RNAiMAX Reagent (Thermo Fisher Scientific) following the manufacturer’s procedures to create a mimic-lipid complex in Opti-MEM Medium (all reagents were from Thermo Fisher Scientific). The complex was added dropwise to each well with a final molarity of 20 nM. Cells were incubated for 6 hours and then the media was changed with fresh complete media. At 72 hours after transfection, cells were harvested for downstream analyses.

### Bulk RNA-seq and pathway analysis.

Healthy lung ECs were transfected with control and miR-205-5p mimics. Three days after transfection, total RNA was isolated using the RNeasy Micro Kit (Qiagen), following the manufacturer’s instructions. RNA samples with RIN values >6 were approved for library preparation and sequencing. RNA libraries were prepared using 200 ng of total RNA. The library was validated with a 2100 Bioanalyzer (Agilent Technologies) to determine the size distribution and concentration, and sequencing was performed with a NextSeq 2000 (Illumina) at the Boston University Microarray and Sequencing Resource core facility.

FASTQ files were aligned to human genome build hg38 using STAR (version 2.7.9a). Ensembl-Gene-level counts for nonmitochondrial genes were generated using featureCounts (Subread package, version 1.6.2) and Ensembl annotation build 112 (uniquely aligned proper pairs, same strand). Variance-stabilizing transformation (VST) was accomplished using the DESeq2 R package (version 1.23.10). PCA was performed using the prcomp R function with VST-normalized expression values that were then *z* score–normalized (set to a mean of 0 and a SD of 1) across all samples within each gene. Differential gene expression was assessed using raw count data with the Wald test implemented in the DESeq2 R package. Correction for multiple hypothesis testing was accomplished using the Benjamini-Hochberg FDR. All analyses were performed using the R environment for statistical computing (version 4.1.2). Pathway analyses were carried out using differentially expressed genes with IPA software (QIAGEN). Heatmaps were created using Prism 9 (GraphPad Software) using VST values *z* score–normalized across all samples within each gene.

### RNA isolation and qPCR analysis.

Total mRNA was isolated using the RNeasy Micro Kit (Qiagen), followed by Nanodrop concentration and purity analysis. cDNA was synthesized with SuperScript VILO (Thermo Fisher Scientific); RT-PCR was performed using FastStart Essential DNA Green Master (Roche Diagnostics) and analyzed using a QuantStudio3 (Thermo Fisher Scientific). qPCR primers used in this study (Integrated DNA Technologies) are listed in Table 1. miRNAs assays used in this study (Thermo Fisher Scientific) are U6 (assay ID 001973), miR-205-5p (assay ID 477967_mir), and miR-34a-5p (assay ID 478048_mir).

### miR-205-5p inhibition in IPF lung ECs.

IPF lung ECs were plated at 50,000 cells per well of a 12-well plate in 1 mL of complete media Promocell Endothelial Cell Growth Medium MV2 1% Pen/Strep (Promocell). Next day, cells were transfected with mirVana-negative control inhibitor or mir205-5p inhibitor using Lipofectamine RNAiMAX Reagent following the manufacturer’s procedures to create a mimic-lipid complex in Opti-MEM Medium (all reagents were from Thermo Fisher Scientific). The complex was added dropwise to each well with a final molarity of 100 nM. Cells were incubated for 6 hours, and then the media was changed with fresh complete media. At 48 hours after transfection, cells were harvested for RNA isolation and qPCR analysis.

### Ki67 staining.

Human lung ECs (isolated from healthy or IPF lungs; or healthy lung ECs transfected with a control mimic or with a miR-205-5p mimic; or IPF lung ECs transfected with a negative inhibitor control or a miR-205-5p inhibitor) were washed, fixed, and permeabilized in 0.1% Triton X-100 for 5 minutes at room temperature. Cells were then blocked with 4% goat serum for 1 hour, and primary Ki67 (Santa Cruz Biotechnology, sc-23900, 1:100 dilution, RRID: AB 627859) antibody solution was added for an overnight incubation at 4°C. Cells were then washed with PBS and secondary antibody solution of Alexa Fluor 488 goat anti-mouse (Thermo Fisher Scientific, 1:500 dilution, RRID:AB_2534069) and counterstained with DAPI (Biolegend, 422801, 1:1,000 dilution), which was added for 2 hours at room temperature. All antibodies were commercially available and were validated by the respective manufacturers. Fluorescence images of Ki67- and DAPI-stained cells were analyzed using the JaCoP (Just Another Colocalization Plugin) plugin in ImageJ version 1.54g (NIH). Prior to analysis, images were background-subtracted, and thresholds were set to minimize noise. Colocalization between Ki67 and nuclear DAPI signals was then quantified, and Pearson’s correlation coefficient was calculated to assess the degree of nuclear localization of Ki67. At least 10 fields per condition were analyzed to ensure reproducibility.

### SA-β-Gal staining.

Human lung ECs (healthy or IPF-derived; healthy transfected with a control mimic or with a miR-205-5p mimic; IPF transfected with a negative inhibitor control or a miR-205-5p inhibitor) were plated in 8-well chambered slides, and SA-β-Gal staining was performed per the manufacturer’s protocol (Senescence β-Galactosidase Staining kit, Cell Signaling Technology). Briefly, cells were washed with PBS, and 1X fixative solution was added for 15 minutes at room temperature. Fixative was washed away with PBS, and cells were stained with β-Galactosidase staining solution (pH 6.0). Chambers were sealed with a plate cover to prevent evaporation and crystal formation and incubated in the absence of CO_2_ in a dry 37°C incubator for 12 hours. β-Galactosidase staining solution was removed, and cells were rinsed with PBS and stained with DAPI (1:1,000) for 1 hour at room temperature. The chambers were removed, and slides were cover slipped and imaged using an optical microscope and a ×10 objective. For quantification, phase-contrast images of SA-β-Gal staining were first taken, followed by DAPI fluorescent images. Blue-stained cells and total number of DAPI-positive cells were counted, and data were plotted as the percentage of SA-β-Gal–positive cells/total number of cells in each field of view.

### Single-cell RNA-seq and data analysis.

A publicly available human single-cell RNA-seq dataset from Tsukui et al. (GSE132771) (58) was analyzed using the Bio Turing Browser X. CD31^+^ ECs from healthy human lungs and IPF lungs were included in the analysis and are displayed as a violin plot.

### Isolation and testing of EC-derived conditioned medium.

Healthy lung ECs were transfected using mirVana-negative control mimic or miR-205-5p mimic, and IPF lung ECs were transfected using mirVana-negative control inhibitor or miR-205-5p inhibitor (as described above). Media were replaced 6 hours after transfection to remove the transfection reagents, and cells were cultured for 72 hours. Conditioned media from control and miR-205-5p–overexpressing cells was then collected and centrifuged at 300*g* for 5 minutes to remove cellular debris before being applied to human lung fibroblasts. After 2 days or 5 days, RNA isolation and qPCR were performed as described in *RNA isolation and qPCR analysis*. After 5 days, collagen I staining was performed as described in *Immunofluorescence*.

### Target prediction analysis.

The enrichment of genes among predicted targets of the indicated miRNAs was determined by uploading the list of genes downregulated in the miR-205-5p mimic lung ECs experiment into MiRTARGET. Target prediction was carried out using 4 independent tools (miRTARGET, ref. 59; miRWalk, ref. 60; TargetScanHuman, ref. 61; DIANA-MicroT, ref. 62), and target prediction lists were integrated with the list of genes downregulated in miR-205-5p mimic in the lung EC experiment. The resulting gene lists were further integrated, and only downregulated genes predicted to be target by at least 2 bioinformatic tools were included in downstream analysis.

### Statistics.

The individual data points shown in all plots represent data from individual mice or biological replicates from cell culture experiments. Sample size for each experimental group is reported in figure legends. Data are reported as mean ± SD, with statistical comparisons between groups performed using 2-tailed Student’s *t* test or 1-way ANOVA (followed by Tukey’s or Dunnett’s post hoc test). All analyses and plots were generated with GraphPad 10, with statistical significance defined as *P* < 0.05 (GraphPad Prism, RRID:SCR_002798).

### Study approval.

The study was approved by the Loyola University Chicago Institutional Review Board (#216973). Lung tissue was obtained from IPF explanted lungs, from individuals who provided a written informed consent. Healthy control lungs were obtained from deceased donors whose lungs were deemed unsuitable for transplant and were provided by Gift of Hope, Itasca, Illinois, USA (IRB#218010), with consent from family for tissue to be used for research purposes. All animal studies were performed under protocols approved by the Mayo Clinic Institutional Animal Care and Use Committee and Loyola University Chicago’s Institutional Animal Care and Use Committee and conformed to the Animal Research: Reporting of In Vivo Experiments guidelines.

### Data availability.

The RNA-seq data discussed in this publication have been deposited in NCBI’s Gene Expression Omnibus and are accessible through GEO Series accession GSE316481 (https://www.ncbi.nlm.nih.gov/geo/query/acc.cgi?acc=GSE316481). The Supporting Data Values file contains all quantifications performed and shown in plots and is available in the supplemental materials.

## Author contributions

GM and BBR contributed equally to this work and are listed in alphabetical order. GM, BBR, and NC conceived and designed research. GM, BBR, ES, AAR, SH, XTC, and GC performed experiments. GM, BBR, AC, and NC analyzed data. GM, BBR, and NC interpreted data. GM, AC, and NC prepared figures. GM and NC drafted the manuscript. DJT, GL, and NC edited the manuscript. GM, BBR, SH, XTC, GC, ES, AAR, CV, AC, SE, MKG, DJT, GL, and NC approved final version of manuscript.

## Funding support

This work is the result of NIH funding, in whole or in part, and is subject to the NIH Public Access Policy. Through acceptance of this federal funding, the NIH has been given a right to make the work publicly available in PubMed Central.

- NIH Shared Instrumentation Grant 1S10OD034431-01 to Loyola University Chicago.
- NIH grants R01 HL092961 (to DJT), R01 HL166187 (to DJT), and R01 HL158733 (to GL).
- National Cancer Institute Cancer Center Support Grant P30-CA060553, awarded to the Robert H. Lurie Comprehensive Cancer Center, which supports the Northwestern University Mouse Histology and Phenotyping Laboratory (RRID:SCR_017870)

## Supplementary Material

Supplemental data

Supplemental table 1

Supporting data values

## Figures and Tables

**Table 1:**
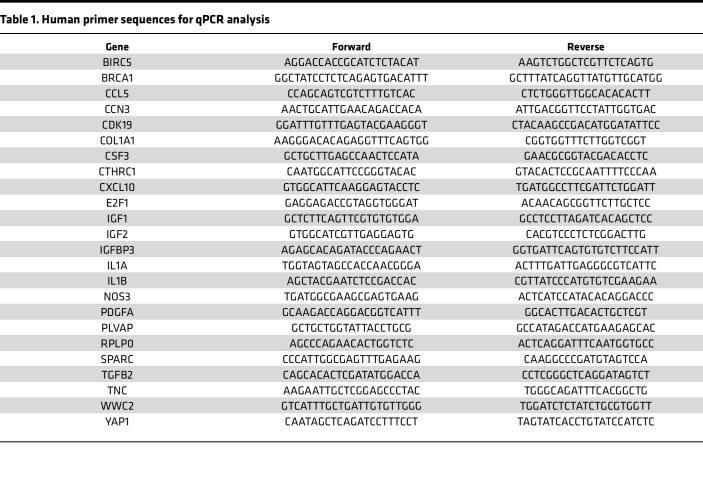
Human primer sequences for qPCR analysis

## Version 1 05/28/2026

In-Press Preview

## Version 2 07/08/2026

Electronic publication
